# Four-Dimensional-Printed Woven Metamaterials for Vibration Reduction and Energy Absorption in Aircraft Landing Gear

**DOI:** 10.3390/ma18143371

**Published:** 2025-07-18

**Authors:** Xiong Wang, Changliang Lin, Liang Li, Yang Lu, Xizhe Zhu, Wenjie Wang

**Affiliations:** 1 Aircraft Design and Research Institute, Harbin Aircraft Industry (Group) Co., Ltd., Harbin 150066, China; 15b918018@hit.edu.cn (X.W.); hf122lcl@163.com (C.L.); 18646170554@163.com (X.Z.); 18846431906@163.com (W.W.); 2 Tianjin Civil Helicopter R&D Branch, Harbin Aircraft Industry (Group) Co., Ltd., Tianjin 300450, China; 3 School of Science, Nanjing University of Science and Technology, Nanjing 210094, China; 4 National Key Laboratory of Helicopter Dynamics, Nanjing University of Aeronautics and Astronautics, Nanjing 210094, China

**Keywords:** mechanical metamaterials, landing gear, 4D printing, energy absorption

## Abstract

Addressing the urgent need for lightweight and reusable energy-absorbing materials in aviation impact resistance, this study introduces an innovative multi-directional braided metamaterial design enabled by 4D printing technology. This approach overcomes the dual challenges of intricate manufacturing processes and the limited functionality inherent to traditional textile preforms. Six distinct braided structural units (types 1–6) were devised based on periodic trigonometric functions (Y = A sin(12πX)), and integrated with shape memory polylactic acid (SMP-PLA), thereby achieving a synergistic combination of topological architecture and adaptive response characteristics. Compression tests reveal that reducing strip density to 50–25% (as in types 1–3) markedly enhances energy absorption performance, achieving a maximum specific energy absorption of 3.3 J/g. Three-point bending tests further demonstrate that the yarn amplitude parameter A is inversely correlated with load-bearing capacity; for instance, the type 1 structure (A = 3) withstands a maximum load stress of 8 MPa, representing a 100% increase compared to the type 2 structure (A = 4.5). A multi-branch viscoelastic constitutive model elucidates the temperature-dependent stress relaxation behavior during the glass–rubber phase transition and clarifies the relaxation time conversion mechanism governed by the Williams–Landel–Ferry (WLF) and Arrhenius equations. Experimental results further confirm the shape memory effect, with the type 3 structure fully recovering its original shape within 3 s under thermal stimulation at 80 °C, thus addressing the non-reusability issue of conventional energy-absorbing structures. This work establishes a new paradigm for the design of impact-resistant aviation components, particularly in the context of anti-collision structures and reusable energy absorption systems for eVTOL aircraft. Future research should further investigate the regulation of multi-stimulus response behaviors and microstructural optimization to advance the engineering application of smart textile metamaterials in aviation protection systems.

## 1. Introduction

Modern weaving technology has not only significantly advanced the production of human clothing but has also played a crucial role in the development of lightweight, fiber-reinforced composite materials [1,2,3,4,5,6,7]. Multidirectional textile structure preforms are commonly employed as reinforcements in composite materials, characterized by the spatial interweaving of yarns, resulting in complex structures [8,9,10,11,12]. Various textile preforming techniques, including weaving, knitting, lamination, braiding, and stitching, are the primary methods for processing these multidirectional textile preforms [13,14,15,16,17]. Notable textile structures, such as three-way woven fabrics, multi-angle laminates, three-dimensional angle interlocking, three-dimensional orthogonal weaves, and three-dimensional braids, are widely utilized in the fabrication of lightweight, high-strength composites due to their superior structural integrity, mechanical properties, and damage tolerance [18,19,20,21,22,23].

Recently, multidirectional topological structures have demonstrated unique potential in aviation impact resistance. Their interwoven networks effectively dissipate stress waves through hierarchical deformation mechanisms, offering innovative solutions for landing gear buffering and fuselage crash-resistant structures [24,25,26,27,28,29,30,31]. However, conventional processing methods for multidirectional textile preforms are complex, labor-intensive, and time-consuming, posing challenges in the manufacturing of critical aviation components. This issue is particularly acute in landing gear systems, as over 65% of aviation accidents involve landing gear failures. Traditional oil–gas buffers, with their fixed stiffness design, lack adaptability to variable landing conditions, while the plastic deformation-based energy absorption of skid-type landing gear is poorly repeatable [32,33,34,35,36,37,38,39,40]. Although multidirectional textile preform-reinforced polymer and ceramic matrix composites exhibit excellent in-plane and out-of-plane mechanical performance and damage tolerance [41,42,43,44,45,46,47], their fabrication remains constrained by the complexity of traditional molding processes, limited design flexibility of weaving equipment, and difficulties in functionalizing the preforms.

In contrast, 3D printing technology offers advantages such as streamlined processes, reduced time and material consumption, and greater structural topology flexibility, presenting a novel approach for manufacturing multidirectional textile structure preforms [48,49,50,51]. In aviation impact resistance, the demand for lightweight, multifunctional integration has become increasingly critical. Traditional lattice structures, which rely on plastic deformation for energy absorption, are not reusable. However, woven metamaterials produced via additive manufacturing overcome this limitation. These structures leverage multilevel topological designs to dissipate energy through various mechanisms, including elastic deformation and fiber slip, enabling adaptive switching between energy absorption modes in bird strike scenarios (101 m/s) and crash scenarios (100 m/s) [52,53,54]. This capability introduces a new paradigm for crash resistance in emerging aircraft, such as eVTOL.

The development of 3D printing technology has stimulated extensive research on multidirectional textile preforms and their composite reinforcements. Quan et al. [52,55,56] employed fused deposition modeling (FDM) to print acrylonitrile–butadiene–styrene (ABS) filaments, fabricating complex multidirectional preforms such as plain weave, corner interlocking, orthogonal, and woven structures. Transformations based on Cartesian, cylindrical, and spherical coordinates enable the topological design of three-dimensional orthogonal structures [1,2,14,57], which are challenging to achieve with traditional textile molding methods. However, mechanical testing indicates that the compressive performance of 3D-printed chopped carbon fiber/ABS preforms is inferior to that of pure ABS preforms, primarily due to increased void content within the fiber-reinforced samples [27]. Tian et al. [25] investigated the compressive properties of 3D-printed woven preforms infused with different resins, demonstrating that the interfacial bonding between the printed preform and the resin significantly affects the mechanical performance of the composite. Rahmatabadi et al. applied post-heat treatment for the first time to improve the stress recovery of short carbon fiber-reinforced PETG (SCFRPETG). By performing heat treatment, the shape memory effect of PETG composites was significantly improved [58]. The polylactic acid/starch biocomposites studied by Baniasadi et al. and Mahyar et al. also provide more possibilities for the application and promotion of 3D printing [59,60].

Multidirectional textile structure preforms and their composites are characterized by robust structural integrity and excellent damage tolerance. Currently, functional textile-based devices are extensively applied in sensors [61], supercapacitors [62], energy harvesting systems [63,64,65], actuators [66], and related areas. Recent research has begun to explore their potential in aviation impact resistance, where bionic woven structures, produced through 4D printing technology, demonstrate shape memory effects. These structures can fully recover their original shapes upon thermal activation after impact, substantially enhancing the reusability of energy-absorbing components [67]. Sanei [68] conducted preliminary investigations into the design, fabrication, and mechanical properties of 3D-printed multidirectional textile preforms, demonstrating that 3D printing allows for the creation of more complex and irregular preform orientations. However, the use of engineering plastic ABS, which lacks multifunctional capabilities, limits its broader application. To address this, Zhao et al. [69] employed 4D-printed shape memory polymers to fabricate circular tube braided structure preforms and analyzed their shape memory properties. They found that preforms with fewer layers and larger braiding angles exhibited faster recovery.

Despite these advancements, the application of 4D-printed multidirectional textile structure preforms remains underexplored, particularly concerning microstructural characterization, optimization, shape recovery performance enhancement, and multi-stimulus shape recovery behavior. Therefore, this study will focus on the shape recovery performance and optimization strategies of 4D-printed braided structure preforms and their composites, aiming to advance their application in aviation impact resistance.

## 2. Design and Fabrication of the Metamaterials

**Figure 1:**
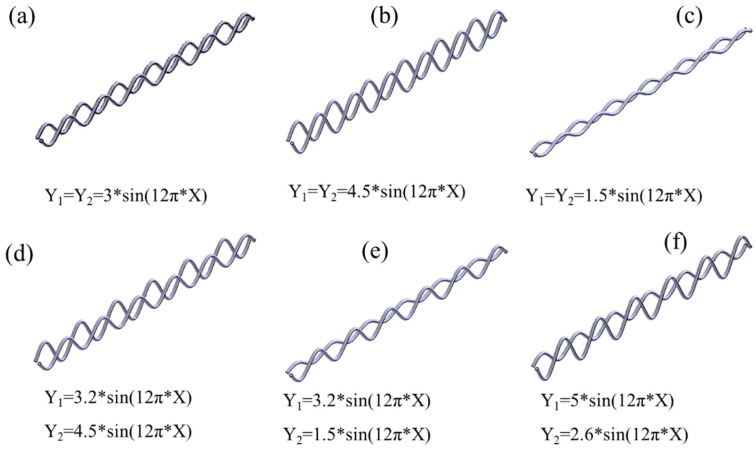
Design of braided structure unit cells with different design parameters: (**a**) Type 1, (**b**) Type 2, (**c**) Type 3, (**d**) Type 4, (**e**) Type 5, and (**f**) Type 6.

**Figure 2:**
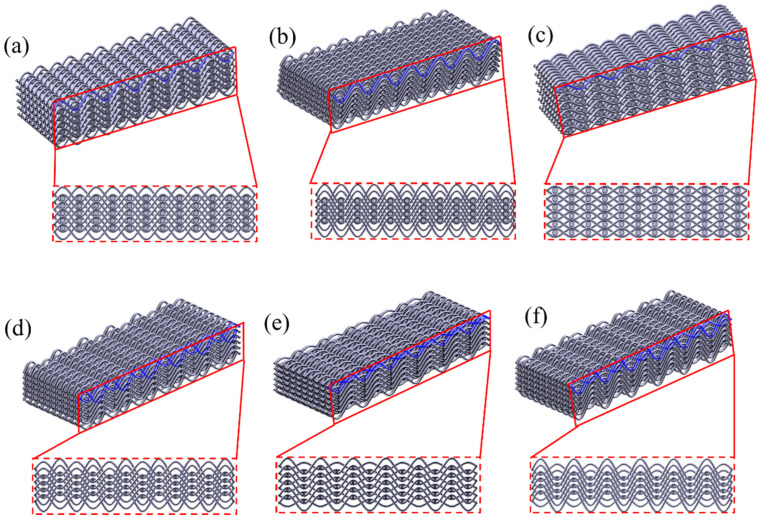
Braided structures with different design parameters: (**a**) Type 1, (**b**) Type 2, (**c**) Type 3, (**d**) Type 4, (**e**) Type 5, and (**f**) Type 6.

**Figure 3:**
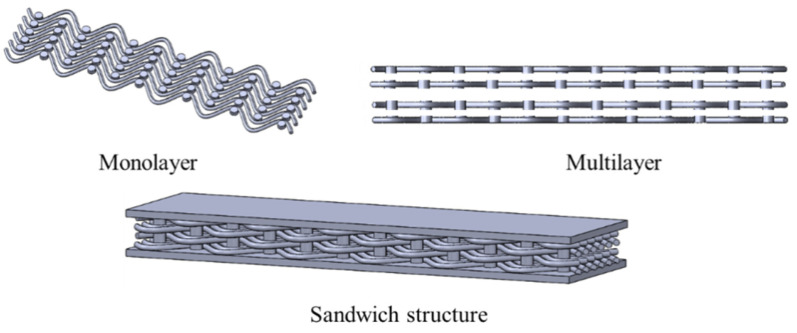
Three-dimensional image of woven structural mechanics metamaterial.

**Figure 4:**
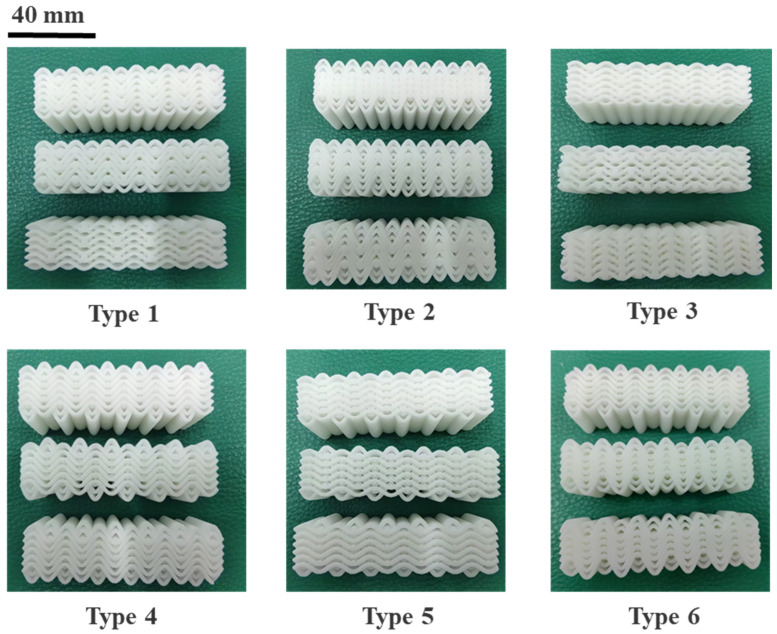
Optical image of 4D printed woven metamaterial specimen.

The design parameters of the structure are shown in Table 1, where the shape of the yarn is configured according to the periodic trigonometric function Y = A*sin(12π*X) (A represents the amplitude of the yarn, and X is the lateral position). Figure 1 and Figure 2 show the unit cell design of the woven structure with different design parameters. Figure 3 shows three-dimensional image of woven structural mechanics metamaterial. Figure 4 shows the optical image of the woven structure mechanical metamaterial. Three specimens of each type were prepared, representing different bar densities (100%, 50%, and 25%).

This project uses Catia (2021) design software for 3D modeling of the woven structure, and ultimaker Cura 5.7.1 slicing software for slicing. The 3D printing equipment uses Bambu A1 3D printer (Shenzhen Bambu Technology Co., Ltd. (Bambu Lab), Shenzhen, China). The printing consumables are: Anycubic PLA+. The nozzle diameter is 0.4 mm.

## 3. Mechanical Properties of SMP-PLA

### 3.1. Uniaxial Tensile Test of Shape Memory Materials

**Figure 5:**
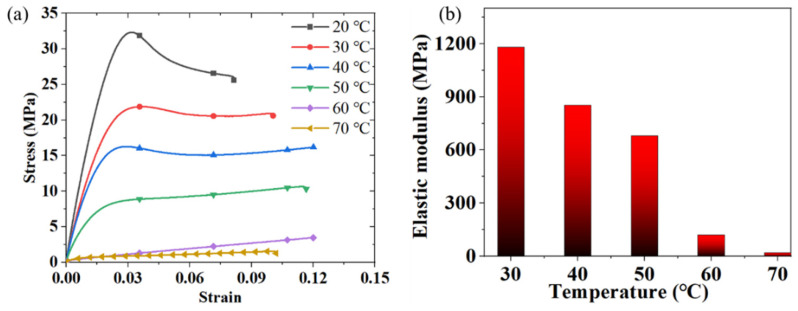
Mechanical properties of PLA-SMP: (**a**) uniaxial tensile test at room temperature, and (**b**) elastic modulus at different temperatures.

The material used to prepare the structure is a shape memory material. The dumbbell-shaped specimen (ASTM D638 [70]: 115 mm × 6 mm × 2 mm) was subjected to a room temperature uniaxial tensile test on a Zwick-010 tensile machine (ZwickRoell, Ulm, Germany) with an environmental chamber at a test rate of 2 mm/min. Figure 5a shows the stress–strain curves of PLA-SMP under uniaxial tension at different temperatures. The material first undergoes an elastic stage, and after being stretched to a strain of 0.03, it begins to undergo plastic deformation until it breaks. When the test temperature exceeds 60 °C, no obvious plastic deformation is exhibited. As shown in Figure 5b, the Young’s modulus of PLA-SMP at room temperature is 1200 GPa, the strength is 33 MPa, and the elongation at break is 9%.

### 3.2. Relaxation Test of Shape Memory Materials

**Figure 6:**
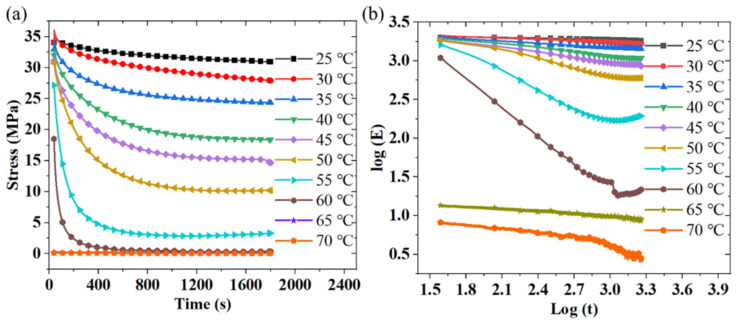
Changes in relaxation modulus of materials at different temperatures over time: (**a**) stress relaxation test of PLA-SMP at different temperatures, (**b**) time–temperature equivalent master curve.

The stress relaxation behavior of PLA-SMP at different temperatures (25 °C, 30 °C, 35 °C, 40 °C, 45 °C, 50 °C, 55 °C, 60 °C, 65 °C, 70 °C, and 75 °C) was characterized on a Zwick-010 stretching machine with an environmental chamber. In the relaxation test, the sample was stretched after being stationary at the target temperature for 30 min, then maintained in deformation for 1800 s, and the decay of the force value was recorded. Figure 6a shows the change in relaxation modulus over time at different temperatures. As the temperature increases, PLA-SMP transforms from the glass phase to the rubber phase, resulting in a decrease in the initial relaxation modulus with increasing temperature. It is worth noting that at 65 degrees Celsius and 70 degrees Celsius, the relaxation modulus and time curves of the material almost coincide. Figure 6b shows the time–temperature equivalent master curve of the PLA-SMP. As the temperature increases, PLA-SMP transforms from the glass phase to the rubber phase, resulting in a decrease in the initial relaxation modulus with increasing temperature.

### 3.3. Constitutive Theory

**Figure 7:**
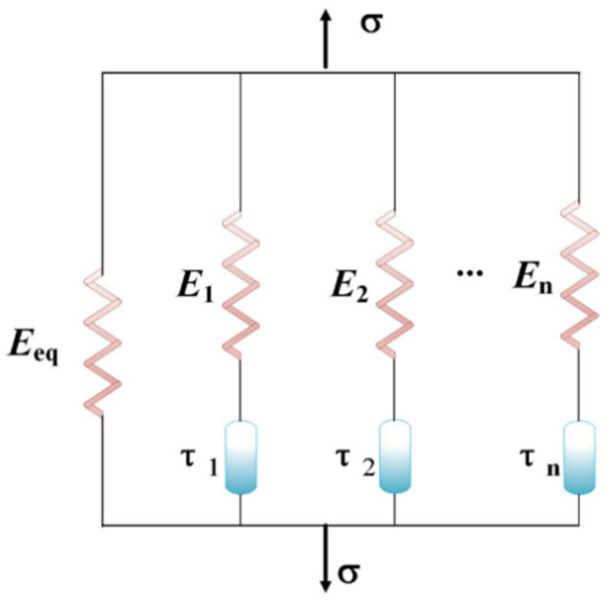
Schematic diagram of multi-branch model.

The multi-branch constitutive model can be used to describe the viscoelastic deformation behavior of PLA-SMP. Figure 7 shows a schematic diagram of the multi-branch constitutive model, which consists of a spring and multiple Maxwell models in parallel, representing the equilibrium behavior and non-equilibrium behavior of SMP, respectively. The equilibrium branch of the model consists of a spring, and the non-equilibrium branch consists of multiple Maxwell models in parallel. Among them, the Maxwell model consists of a spring and a viscoelastic pot in series.

According to the Boltzmann superposition principle, the relationship between total stress and strain can be obtained as

(1) $$\sigma \left(t\right)=E_{eq}\varepsilon_{m}\left(t\right)+\sum_{i=1}^{n}E_{i}\varepsilon_{i}^{e}$$

in which the first term on the right side of the equation represents the stress–strain relationship of the equilibrium branch, and the second term on the right side of the equation represents the contribution of each non-equilibrium branch to the total stress.

(2) $$\varepsilon_{i}^{e}=\int_{0}^{t}\frac{d\varepsilon_{m}\left(s\right)}{dt}\exp\left(-\int_{s}^{t}\frac{dt^{\prime }}{d\tau_{i}\left(T,t^{\prime }\right)}\right)ds$$

where $\varepsilon_{m}$ represents the total strain, $E_{eq}$ and $E_{i}$ represents the modulus of the equilibrium branch and the non-equilibrium branch, respectively, and *n* represents the number of non-equilibrium branches. $\tau_{i}$ represents the relaxation time, which has temperature-dependent characteristics and follows the following rule:

(3) $$\tau_{i}=\alpha_{T}\left(T\right)\tau_{i}^{0}$$

where $\tau_{i}^{0}$ represents the relaxation time at the reference temperature, $\alpha_{T}\left(T\right)$ represents the time–temperature superposition (TTSP) shift factor. When the temperature is lower than the shifting temperature (Ts), $\alpha_{T}\left(T\right)$ satisfies Arrhenius-type behavior

(4) $$\ln\alpha_{T}\left(T\right)=-\frac{AF_{c}}{k_{b}}\left(\frac{1}{T}-\frac{1}{T_{g}}\right)$$

where *A* is the material constant, $F_{c}$ is the configuration energy, $k_{b}$ and represents the Boltzmann constant. When the temperature is close to $T_{s}$ or above, the Williams–Landel-Ferry (WLF) equation is used to calculate $\alpha_{T}\left(T\right)$: [69,71]

(5) $$\log\alpha_{T}\left(T\right)=-\frac{C_{1}\left(T-T_{ref}\right)}{C_{2}+\left(T-T_{ref}\right)}$$

where $T_{ref}$ is the reference temperature, $C_{1}$ and $C_{2}$ are material constants.

The relaxation modulus of a material can be written in the form of a Prony series:

(6) $$E\left(t\right)=E_{eq}+\sum_{i=1}^{n}E_{i}e^{\left(-\frac{t}{\tau_{i}}\right)}$$

By translating the relaxation modulus curves at different temperatures, the TTSP translation factor and stress relaxation master curve can be obtained.

## 4. Mechanical Properties of Metamaterials

### 4.1. Compressive Test

**Figure 8:**
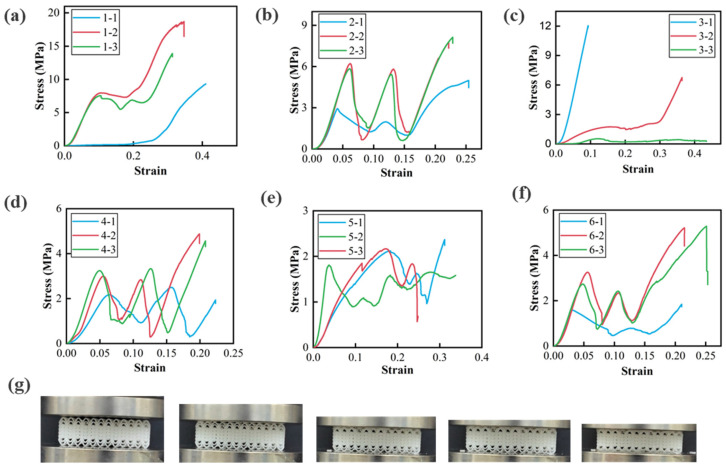
Compression test results of woven metamaterials: (**a**) Type 1, (**b**) Type 2, (**c**) Type 3, (**d**) Type 4, (**e**) Type 5, (**f**) Type 6, (**g**) optical image of compression test.

Compression tests were conducted on braided structural mechanical metamaterials with varying geometric parameters using a Zwick-010 tensile machine at a loading rate of 2 mm/min to evaluate their compressive deformation behavior. As shown in Figure 8, a decrease in bar density reduces ligament stiffness, leading to a lower initial equivalent stiffness of the metamaterial. Under increasing compressive loads, the top yarn ligament bends first, causing an initial rise in stress. As loading continues, the top yarn ligament becomes compacted, followed by progressive bending and deformation of the bars embedded within the yarn. This process results in a fluctuating or plateau stage in the stress–strain curve (Figure 8a,b,d–f). Deformation primarily occurs in the surface yarn ligament and inner bars, while the inner yarn ligament remains largely undeformed. Due to stress concentration and ligament contact/extrusion, the maximum principal strain is observed at the ligament inflection points (Figure 8g).

As illustrated in Figure 8c, increasing bar density enhances yarn ligament stiffness, thereby raising the initial equivalent stiffness of the metamaterial. However, excessively high bar density negatively impacts energy absorption capacity. This occurs because insufficient bending space for the yarn ligament forces the applied load onto the bars, preventing the formation of an energy absorption plateau. For instance, the stress–strain curves of the Type 3-1 and Type 3-2 structures lack a distinct plateau region.

The yarn curve equation significantly influences metamaterial performance. When Type 2, Type 4, Type 5, and Type 6 structures undergo compression, their stress–strain curves exhibit pronounced fluctuations. This behavior arises from differences in the amplitudes (*A*) of Y1 and Y2 in the yarn curve equation, causing the yarn ligament with a larger *A* to deform and fail first. Once fully compressed, the ligament with a smaller *A* continues to bear the load. Additionally, due to the repetitive arrangement of yarn ligaments along the longitudinal direction, the stress–strain curve typically features two to three maximum stress peaks.

A comparison of the compressive stress–strain curves of Type 1, Type 2, Type 4, Type 5, and Type 6 metamaterials reveals a significant influence of bar diameter on structural stress. In Type 1 and Type 2 structures, where the bar diameter is 2.1 mm, the average stress in the plateau stage is 8 MPa and 4 MPa, respectively. In contrast, the bar diameter in Type 4, Type 5, and Type 6 structures is 0.8 mm, resulting in average plateau stresses of 2 MPa, 1 MPa, and 2 MPa, considerably lower than that of the Type 1 metamaterial. These findings highlight the critical role of bar diameter in determining the mechanical performance of braided structural mechanical metamaterials.

### 4.2. Three-Point Bending Test

Three-point bending tests were conducted using a Zwick/Roell Z10 (ZwickRoell, Ulm, Germany) universal testing machine equipped with a bending fixture, operating at a loading rate of 1 mm/min. The bending specimens were designed in accordance with ASTM D7264/D7264M-15 [72], with nominal dimensions of 85 mm × 13 mm × 2.4 mm.

**Figure 9:**
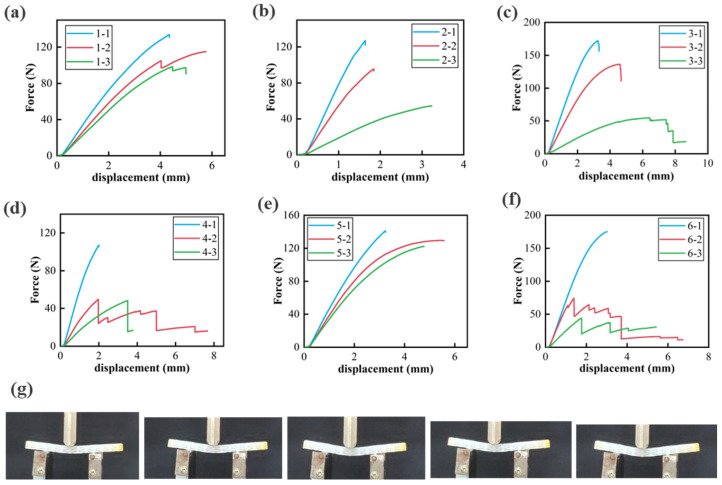
Three-point bending test results of woven metamaterials: (**a**) Type 1, (**b**) Type 2, (**c**) Type 3, (**d**) Type 4, (**e**) Type 5, (**f**) Type 6, (**g**) optical image of three-point bending compression test.

As shown in Figure 9, the force–displacement response exhibits an initial linear regime, corresponding to the elastic deformation stage of the metamaterial. Beyond a certain displacement threshold, the load–displacement curve deviates from linearity, indicating the onset of plastic yielding. Analysis of specimens of the same type reveals that the bending modulus in the linear regime increases with bar density (Figure 9a). Furthermore, as illustrated in Figure 9b–f, an examination of different woven metamaterial structures shows that a reduction in the amplitude of the yarn ligament curve equation (e.g., A_type1_ = 3, A_type2_ = 4.5, A_type3_ = 1.5) leads to an increase in the maximum bending load within a certain range (F_max, type1_ = 140 N, F_max, type2_ = 120 N, F_max, type3_ = 180 N). This effect arises because structures with larger amplitudes exhibit sharper peak and valley angles, which increase stress concentration during bending, making them more prone to failure. Consequently, these structures exhibit reduced bending displacement and a lower maximum load capacity.

Following peak load, the load–displacement curve declines, indicating plastic deformation and, ultimately, material failure. Some curves exhibit a steep drop without a pronounced plastic region, suggesting brittle fracture upon reaching the critical load. In contrast, others display a more gradual decline with a serrated pattern (e.g., 3-3, 4-2, 6-2, 6-3), indicative of non-uniform fracture propagation within the material. This behavior suggests that the metamaterial retains a degree of ductility, allowing it to accommodate some deformation prior to failure. For instance, in group D, the green curve corresponding to sample 4-3 exhibits a sharp drop, signifying brittle failure, whereas sample 4-2 shows a more gradual, tortuous descent, indicating enhanced plasticity.

The bar density in woven metamaterials exhibits a nonlinear relationship with the plastic properties of the material. Notably, sample 4-2 demonstrates a more extended plastic stage compared to 4-1 and 4-3, while 5-2 similarly exhibits a longer plastic stage relative to 5-1 and 5-3. These findings suggest that an optimal bar density range should be considered in structural design to enhance mechanical performance while minimizing weight.

### 4.3. Energy Absorption Performance Characterization

To further characterize the energy absorption characteristics of the 4D-printed woven metamaterial, using the load–displacement curves obtained during quasi-static compression of the woven metamaterial, the total energy absorption (EAs) and specific energy absorption (SEA) can be calculated by the following equations:

(7) $$EA=\int_{0}^{\delta_{0}}F(\delta )d\delta$$

(8) $$SEA=\frac{EA}{M}$$

where *EA* is the total energy absorption, δ and *F* represent deformation and load, respectively, and $\delta_{0}$ is the densification deformation; *SEA* is the specific energy absorption, *M* is the mass of the braided structural mechanics metamaterial.

**Figure 10:**
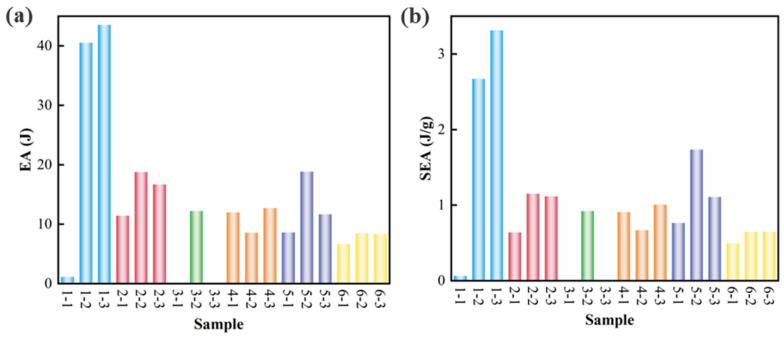
(**a**) Compression energy absorption of woven structural mechanical metamaterials, (**b**) compression specific energy absorption of woven structural mechanical metamaterials.

Figure 10a,b, respectively, show the *EA* and *SEA* of mechanical metamaterials with woven structural configurations. In general, the energy absorption capacity of mechanical metamaterials with type 1 structure is much stronger than that of other types. Among them, woven structural mechanical metamaterials 1-3 have the largest specific energy absorption, which is 3.3 J/g. The woven structural mechanical metamaterials with bar densities of 50% and 25% have higher EA and SEA than those with a bar density of 100%. In addition, for woven structural mechanical metamaterials 1-1, 3-1, and 3-3, the displacement–load relationship of the metamaterial under compression load is “J” shaped, which makes it have a lower energy absorption capacity. Compared with other configurations, woven structural mechanical metamaterials with type 1, type 2, and type 3 configurations have stronger compression deformation capabilities. Therefore, among the tested specimens, woven structural mechanical metamaterials with 1-2 and 1-3 structures have higher energy absorption capabilities.

### 4.4. Reconfigurable Performance Characterization

In this section, we evaluate the shape memory properties of 4D-printed braided metamaterials by numerical simulation and experimental methods. The deformation process of the braided metamaterial is simulated and analyzed by the general Maxwell model combined with the WLF time–temperature equivalent relationship. The geometric model of the woven metamaterial was constructed using Solidworks software (version 2023), and then imported into the ABAQUS (version 2023) commercial finite element platform for simulation calculations. Specifically, the BTS-I bionic tracheal stent is meshed with 36,551 quadratic tetrahedral elements (C3D10) and 503 solid eight-node tetrahedral elements (C3D8R), while the BTS-II stent is meshed with 28,509 quadratic tetrahedral elements (C3D10) and 503 C3D8R elements. The mechanical behavior under a high-temperature environment is described by the Neo-Hookean constitutive model; for the response in the viscoelastic stage, the time–domain viscoelastic model combining the Prony series and the WLF equation is used. The relevant material parameters are listed in Table 1. The experimentally obtained stress relaxation data were used as shear test input and imported into the viscoelastic material module of ABAQUS to realize the simulation.

**Figure 11:**
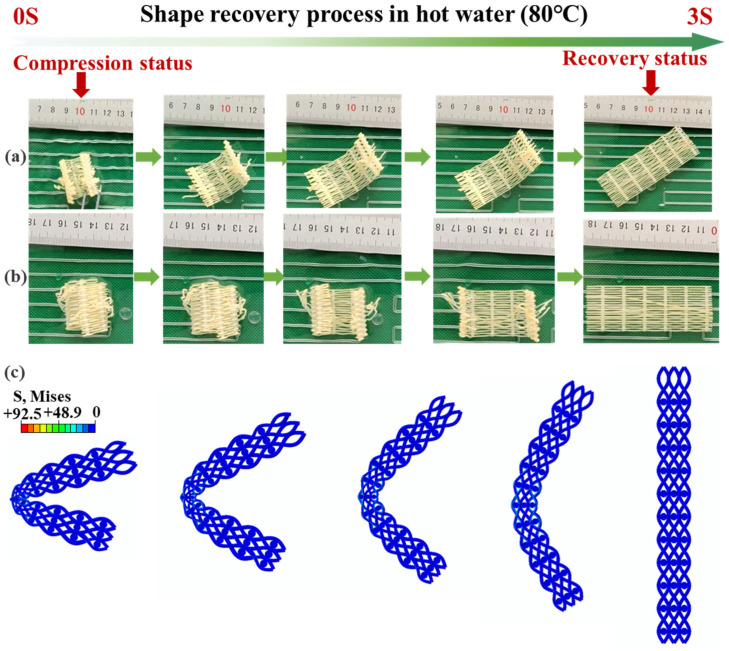
Braided structural mechanics metamaterial shape recovery test: (**a**) The process of the shape recovery experiment of the 4D-printed braided metamaterial after folding, (**b**) the process of the shape recovery experiment of the 4D-printed braided metamaterial after curling, (**c**) the finite element calculation results of the shape recovery of the 4D-printed braided metamaterial after folding.

Figure 11 shows the experimental and simulation processes of the shape memory recovery behavior of the woven structure mechanical metamaterial made of PLA-SMP, respectively. The metamaterial can remember its temporary shape and return to its original shape after heating again. Figure 11a shows the realization process of the braided mechanical metamaterial after folding in half, and Figure 11b shows the realization process of the braided mechanical metamaterial after curling and unfolding. The results show that even though the specimen is completely curled or folded in half, it can recover to its original shape in 80 °C hot water, with a recovery time of only 3 s and a recovery rate of 100%. The results show that the specimen can recover to its original shape in hot water at 80 °C, and the recovery time is only 3 s, with a recovery rate of 100%. Figure 11c shows the finite element calculation results of the braided mechanical metamaterial recovering to its original shape when folded, which is highly consistent with the experimental results.

## 5. Conclusions

Addressing the urgent demand for lightweight and reusable energy-absorbing materials in aviation impact resistance, this study introduces a novel approach for fabricating multi-directional braided metamaterials using 4D printing technology. This method overcomes the dual limitations of complex processing and the limited functionality of conventional textile preforms. Six types of braided structural units (Types 1–6) were designed based on periodic trigonometric functions, leveraging shape-memory PLA to integrate topological architecture with intelligent responsiveness.

Experimental results reveal that under compressive loading, the metamaterial exhibits a staged deformation mechanism. When bar density decreases to 50–25% (e.g., Type 1-2/3), energy absorption capacity significantly improves, with a maximum specific energy absorption of 3.3 J/g. Additionally, three-point bending tests demonstrate a negative correlation between yarn amplitude and load-bearing capacity. Specifically, the Type 1 structure, with an amplitude of A = 3, achieves a maximum load stress of 8 MPa, 100% higher than that of Type 2 (A = 4.5). Notably, the material exhibits an exceptional shape-memory effect, achieving full shape recovery within three seconds under thermal stimulation at 80 °C. By dissipating energy through a multi-level deformation mechanism, this metamaterial addresses the limitations of traditional energy-absorbing structures, which are typically non-reusable.

This study provides valuable insights for the design of crash-resistant aerospace structures and reusable energy-absorbing components, contributing to advancements in impact-resistant materials.

## Figures and Tables

**Table 1:** Design parameters of braided structural mechanics metamaterial.

Type	Yarn 1	Yarn 2	Diameter of the Bar (mm)	Overall Size (mm)
1	3	3	2.1	80 × 29.5 × 22
2	4.5	4.5	2.1	80 × 29.5 × 25
3	1.5	1.5	2.1	80 × 29.5 × 18
4	3.2	4.5	0.8	80 × 29.5 × 25
5	3.2	1.5	0.8	80 × 29.5 × 21
6	5	2.6	0.8	80 × 29.5 × 24

## Data Availability

The raw data supporting the conclusions of this article will be made available by the authors on request.
